# Virtual reality-based Mindfulness-Oriented Recovery Enhancement (MORE-VR) as an adjunct to medications for opioid use disorder: a Phase 1 trial

**DOI:** 10.1080/07853890.2024.2392870

**Published:** 2024-08-22

**Authors:** Eric L. Garland, Marc Recasens, Bayley J. Taple, Gary W. Donaldson, Risa B. Weisberg

**Affiliations:** a Center on Mindfulness and Integrative Health Intervention Development, College of Social Work, University of Utah, Salt Lake City, Utah, USA; b BEHAVR, LLC, DBA RealizedCare, Elizabethtown, Kentucky, USA; c Department of Psychiatry & Behavioral Sciences, Northwestern University Feinberg School of Medicine, Chicago, Illinois, USA; d Pain Research Center, Department of Anesthesiology, University of Utah, Salt Lake City, Utah, USA; e Department of Psychiatry, Chobanian and Avedisian School of Medicine, Boston University, Boston, Massachusetts, USA

**Keywords:** MORE-VR, virtual reality, opioid use disorder, feasibility, craving, affect

## Abstract

**Introduction:**

Medications for opioid use disorder (MOUD) are the most effective interventions for this condition, yet many patients discontinue treatment. Though adjunct psychosocial treatments are recommended to increase retention and reduce relapse, the scarcity of trained providers hinders access to and utilization of evidence-based interventions. We conducted a Phase 1 study to assess the feasibility of a virtual reality-delivered Mindfulness-Oriented Recovery Enhancement (MORE-VR) intervention for patients receiving MOUD.

**Patients and Methods:**

Patients receiving buprenorphine or methadone for OUD (*N* = 34) were scheduled for 8 weekly sessions of MORE-VR. Enrollment and retention rates were analyzed. Participants reported on the usability and acceptability of MORE-VR, opioid use, and craving and affect before and after each VR session. Heart rate was monitored during one session of MORE-VR.

**Results:**

Twenty-three participants completed four or more MORE-VR sessions (minimum recommended intervention dose). Participants reported high usability and acceptability of MORE-VR, which had an excellent safety profile. Illicit opioid use decreased significantly from pre- to post-treatment (*F* = 4.44, *p*=.04). We observed a significant within-session decrease in opioid craving (*F* = 39.3, *p*<.001) and negative affect (*F* = 36.3, *p*<.001), and a significant within-session increase in positive affect (*F* = 23.6, *p*<.001). Heart rate shifted during cue-exposure and mindfulness practices (*F* = 6.79, *p*<.001).

**Conclusions:**

High retention, usability and acceptability rates and low adverse events demonstrated that MORE-VR is a feasible, engaging, and safe intervention. Our findings show that MORE-VR can be delivered as an adjunctive intervention to MOUD and suggest that MORE-VR may improve OUD treatment outcomes and modulate autonomic responses. MORE-VR’s efficacy will be tested in a subsequent Phase 2 trial.

**Trial Registration:**

ClinicalTrials.gov NCT05034276; https://classic.clinicaltrials.gov/ct2/show/NCT05034276

## KEY MESSAGES

- MORE-VR is a digital therapeutic that uses Virtual Reality to deliver an 8-week mindfulness-based intervention for opioid use disorder treatment.
- Patients with OUD reported high completion rates, usability and acceptability.
- In participants receiving MORE-VR as an adjunct to MOUD, reduced craving and opioid use was reported over time.

## 1. Introduction

Approximately 16 million individuals worldwide and three million US citizens have opioid use disorder (OUD) [1]. In the US, opioid overdoses kill nearly 70,000 people every year [2], and OUD exacts an estimated annual cost of $56 billion dollars in the US alone [3].

Medications for opioid use disorder (MOUD), such as methadone, buprenorphine, or naltrexone, are currently the most effective intervention for OUD, with demonstrated effects on mortality, treatment retention, and remission [4,5]. Yet, 42% of people who begin MOUD discontinue it within 24 weeks [6] and 50% of people retained in MOUD have a relapse within six months [7]. MOUD is often delivered with adjunctive psychosocial interventions as part of medication assisted treatment, including counseling, peer support, and contingency management [8–10]. However, studies show limited additive benefit of standard psychosocial therapies for addiction [11,12]. As such, novel adjunctive behavioral interventions are needed to improve MOUD treatment outcomes.

Mindfulness-based interventions (MBIs) have been highlighted as promising adjunctive treatments for substance use disorders [13–16]. Among MBIs, Mindfulness-Oriented Recovery Enhancement (MORE) has garnered the strongest evidence base as an intervention for opioid misuse and OUD. MORE unites training in mindfulness, reappraisal, and savoring to target addiction and its comorbidities [17]. MORE aims to address the hedonic dysregulation in brain reward systems underpinning addictive behaviors [18,19]. Thus, MORE may target key reward deficits in addiction that are not currently treated by MOUD. Attentional, autonomic, electroencephalographic (EEG), and functional neuroimaging markers show that MORE is associated with reduced drug cue-reactivity and a strengthening of natural reward responses [19–24]. MORE’s efficacy as a treatment for OUD and opioid misuse is supported by multiple randomized clinical trials (RCTs) and meta-analyses [16,25]. For instance, a recent full-scale RCT (*N* = 250) found that MORE reduced the occurrence of opioid misuse at nine month follow-up by 45%, doubling the odds of reduced opioid misuse in the supportive psychotherapy control group [26]. In a pilot RCT, MORE plus MOUD was associated with significantly fewer days of drug use and less craving than usual MOUD treatment [27]. These findings were recently replicated in another RCT (*N* = 154), which again showed that MORE plus MOUD was associated with significantly fewer days of drug use, a lower risk of relapse, and greater MOUD adherence and retention than usual MOUD treatment [28].

While the efficacy of the face-to-face MORE intervention has now been demonstrated [25], MORE is resource intensive, requiring significant human interaction and training to deliver, thus limiting its accessibility and scalability. Providing MORE *via* virtual reality (MORE-VR), has been proposed as a solution to deliver MORE with high fidelity while addressing barriers and improving access to this evidence-based intervention. Virtual reality (VR) is particularly suited to deliver MORE because of its power as a virtual exposure engine [29,30]. Exposure to drug cues through VR can reliably elicit drug cue-reactivity [30–33], and cue-exposure therapy through VR has been shown to decrease craving [34–36]. In addition to its situational modeling capabilities, VR can provide controlled and graded immersive exposures for the patient to test and utilize their newly learned skills and facilitate retrieval-extinction training, which has been shown to reduce drug use [37,38]. Ultimately, self-guided digital therapeutics like MORE-VR may lead to enhanced patient engagement, improved treatment outcomes, and reduced healthcare costs.

This is the first study of a project aimed at developing and evaluating MORE-VR and, to our knowledge, the first study examining the impact of a multi-session VR-delivered MBI for OUD. The primary aim of the present study was to assess MORE-VR’s feasibility, engagement, acceptability, usability and safety in patients receiving MOUD. An additional study aim was to obtain an initial indication of MORE-VR’s therapeutic and physiological impact.

## 2. Patients and methods

### 2.1. Design and procedure

We used a single-arm, open-label design. MORE-VR sessions occurred once per week over the course of eight weeks. Assessments immediately before and after MORE-VR sessions evaluated within- and between-session craving and affect outcomes. The impact of MORE-VR on opioid use was assessed comparing pre-treatment measures at baseline prior to MORE-VR and post-treatment measures collected after completion of the 8 weekly sessions of MORE-VR. Following Session 8, participants completed a usability and safety assessment (see study procedure timeline in Supplementary Figure 1).

### 2.2. Recruitment and sample

Patients were recruited from October 2021 to January 2022 through electronic health records review, physician referrals, and community advertisements. Inclusion criteria were: (1) ≥18 years old, (2) DSM-5 OUD diagnosis, and (3) current treatment with MOUD. Exclusion criteria were: (1) prior experience with a formal MBI (e.g. Mindfulness-Based Stress Reduction), (2) active psychosis or high risk of suicidality, as assessed with the Mini International Neuropsychiatric Interview (MINI) [39], (3) self-reported or clinically-noted cognitive impairment, and (4) at baseline, unwillingness/inability to remain in MOUD treatment for the duration of the trial. After written consent, study coordinators collected demographic information and outcomes, and scheduled eight-weekly sessions of MORE-VR. Thirty-four participants (mean age = 38.3 years; SD = 9.9 years) with OUD, treated with MOUD, were enrolled in the study.

### 2.3. Intervention

Eight weekly MORE-VR sessions took place in a private room at the Center on Mindfulness and Integrative Health Intervention Development in the University of Utah. A research assistant was present with participants during VR sessions, except when there were ≥ 2 participants present simultaneously. In these cases, the research assistant alternated between rooms to ensure close participant monitoring. The research assistant administered craving and affect assessments before and after the VR sessions and aided in the set-up and launch of the VR session, but did not guide the participant during the VR session. During session 5, which included a drug-cue exposure exercise (see below), the research assistant remained in the room to provide support in the event of a participant reporting high levels of craving or distress. Each session of MORE-VR lasted approximately 45–60 min.

**Figure 1.**
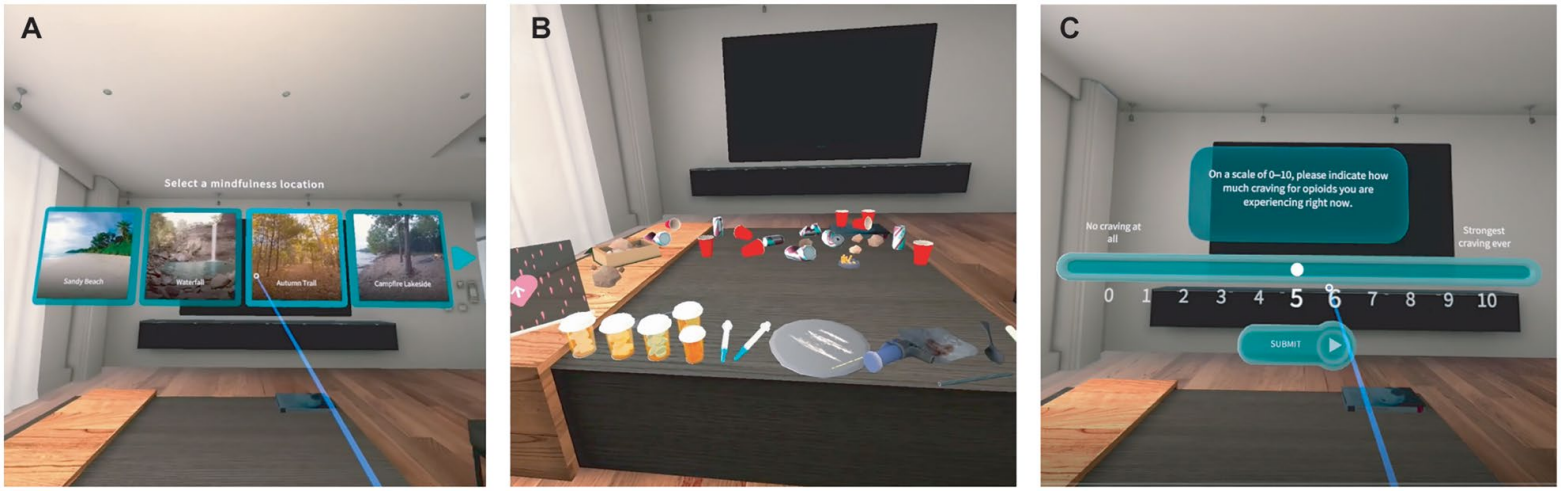
The MORE-VR app: (A) mindfulness location selection menu. (B) Drug cue-exposure exercise. (C) Craving assessment.

MORE-VR was structured in 8 sessions, each providing sequenced training in mindfulness, reappraisal, and savoring skills. Like the face-to-face therapy, each MORE-VR session started with a mindfulness training exercise that consisted of meditation on breathing and bodily sensations to enhance cognitive control over drug craving and provided psychoeducational content to allow for generalization of skill learning. Participants were debriefed about the exercise, and home mindfulness practice assignments. Then, each session focused on a particular topic that defined the psychoeducational content and subsequent mindfulness or therapeutic exercises, including reappraisal (i.e. restructuring) of maladaptive thoughts to downregulate negative emotions, and savoring of pleasurable events and sensations to upregulate positive emotions and amplify natural reward processes (for more details on content of each session, see Supplementary Table 1). Finally, MORE-VR sessions ended with the assignment to engage in 15 min of mindfulness, reappraisal, and savoring practice between sessions as homework. Several notable adaptations of the face-to-face MORE intervention were made to capitalize on the use of VR. In all sessions, mindfulness practices took place in a natural immersive environment selected by the participant (e.g. beach, forest, waterfall) (Figure 1A). Psychoeducation content was presented using 2D animated videos in the virtual environment. Lessons 1 to 8 provided education on the following themes: (1) introducing the concept of mindfulness; (2) automatic habit and addiction; (3) reappraisal of negative emotions; (4) savoring natural rewards and positive emotions; (5) mindfulness of craving; (6) stress and craving; (7) interdependence and meaning in recovery; and (8) relapse prevention. Some sessions leveraged the use of virtual elements, rather than relying on mental visualization as done in face-to-face MORE intervention. For instance, during the therapeutic exercise in session 2, participants were exposed to appetizing and high calorie virtual food items (e.g. ice cream) to elicit craving. During the exercise in session 4, participants practiced savoring by interacting with a virtual rose. Participants could pick up the rose and move it close to their face to appreciate the visual beauty of this virtual object. In session 5, participants engaged in a drug cue-exposure exercise, in which they interacted with drug cues (Figure 1B) to elicit craving before being instructed in a mindfulness of craving technique. Participants’ heart rate (HR) was monitored to assess cue-reactivity during this exercise (details in section 2.3.1). In addition to facilitating drug cue-extinction, this exercise allows for simulated training in therapeutic skill practice in safe, virtual clinically-salient contexts where participants are guided to use MORE skills to address addiction triggers.

#### 2.3.1. Drug cue-exposure and mindfulness of craving exercise

The drug-cue exposure exercise within session 5 had a duration of 20:36 min and was divided into three phases: (1) The exercise began with a 4-minute mindful breathing practice in which participants were asked to focus on sensations of the breath; (2) the second period, between minutes 4 and 6, comprised virtual drug cue-exposure where participants viewed and interacted with virtual drugs and drug paraphernalia (e.g. syringes, opioid pill bottles, bags of heroin, pipes); (3) the third phase, between minute 6 and the end of the exercise, consisted of a mindfulness of craving technique from MORE where participants were first directed to cultivate interoceptive awareness of craving sensations, then to contemplate the negative consequences of indulging in the craving, and finally to cultivate a state of meta-awareness in which cravings were viewed as ephemeral experiences within a larger field of awareness.

### 2.4. Measures

#### 2.4.1. Primary outcomes

Feasibility and engagement: Study feasibility was evaluated by enrollment and retention rates. Enrollment rate was calculated by dividing the number of enrolled participants by the number of patients assessed for eligibility. Retention rate was defined as the ratio of participants completing the minimum intervention dose. The minimum intervention dose of MORE-VR was defined a priori as ≥4 treatment sessions based on treatment completion thresholds established in other mindfulness clinical trials [26,27,40,41].

Acceptability, usability, and safety: Acceptability was evaluated using Likelihood to Recommend (LTR) scales, assessed at the end of each MORE-VR session. In response to the question ‘Please rate how likely you are to recommend this to a colleague or friend?’ participants rated LTR on a 0 to 10 scale (where 0 was ‘not at all’ and 10 was ‘very likely’) presented in the MORE-VR program. LTR scores were used to calculate the Net Promoter Score (NPS). The NPS assesses consumer satisfaction and quality performance [42]. Based on LTR ratings, responses are categorized into three groups: ‘Detractors’ (ratings 0–6), ‘Passives’ (ratings 7 and 8) and ‘Promoters’ (ratings 9 and 10). The overall score is calculated by subtracting the percentage of Detractors from the percentage of Promoters, therefore NPS can range from −100 (i.e. all Detractors) to +100 (i.e. all Promoters). Usability was assessed with the question ‘How easy was it for you to use the virtual reality application today?’ at the end of week 8 on a 0 to 10 scale (where 0 was ‘not at all easy’ and 10 was ‘very easy’). Safety was assessed during post-treatment assessment by asking participants to report adverse events that had occurred during the study.

#### 2.4.2. Secondary outcomes

Days of opioid use: Days of illicit opioid use were assessed with questions from the Addiction Severity Index (ASI) [43]. Participants were asked to report the number of days they used heroin or other opioids without a prescription in the past 30 days. Opioid use was assessed before the start of treatment and then at post-treatment (after the 8^th^ treatment session).

Affect: Positive and negative affect was assessed with the Positive And Negative Affect Scale (PANAS) [44]. The PANAS was measured at the beginning and end of each session. In addition, positive affect was measured with a 0–10 numeric rating scale (NRS) at the beginning and end of each session (where 0 was ‘Not positive at all’ and 10 was ‘Extremely positive’).

Craving: Craving was measured with the Desires for Drug Questionnaire (DDQ) [45]. The DDQ is a 13-item questionnaire for measuring craving with items rated on a 7-point Likert scale. DDQ was completed at the beginning and end of each session. In addition, craving was also measured with a single-item NRS (where 0 was ‘No Craving at All’ and 10 was ‘Strongest Craving Ever’) (Figure 1C).

Heart rate: Participants’ HR was recorded at a sampling frequency of 1 Hz *via* a photoplethysmography-enabled smartwatch integrated in the VR platform, and participants received real-time biofeedback about their HR response to the drug cue-exposure through a graphical icon. Four epochs were created by averaging over periods of 2 min of data: (1) Baseline: using minutes 1–3 of the HR data prior to drug cue-exposure; (2) Cue-exposure: using HR data during minutes 4–6 during the virtual drug cue-exposure activity; (3) Early craving meditation: minutes 7–9 during the mindfulness of craving practice immediately after the cue-exposure (i.e. during mindful interoceptive awareness of craving); and (4) Late craving meditation: minutes 18–20 during the mindfulness of craving practice (i.e. during meta-awareness of the ephemeral nature of craving).

### 2.5. Analysis

Feasibility and engagement, acceptability, usability, and safety outcomes were descriptively analyzed (counts and percentages, means and standard deviations). The present study was focused on feasibility, and thus was not powered to detect a change in proximal outcomes. Yet, the repeated-measures nature of our design afforded comparatively high statistical power. With eight measurement points and a moderate repeated measures correlation (*r*=.30), we estimated a power of .90 to detect a medium effect size for the simple fixed effect of time (Cohen’s *f*=.25) with *N* = 30. We assumed at least a medium effect size based on the large effects of time observed among participants treated with MORE in a prior RCTs for craving [46], and positive affect [47].

A linear mixed model (LMM) assessed changes in days of opioid use between pre- and post-treatment with MORE-VR. This model specified a fixed effect of Time (pre-post). To assess changes in craving and affect across the 8 sessions, a LMM assessed fixed effects of Time (weekly across the 8 sessions), Session (immediately before and after each session, the within-session effect), and the Session X Time interaction. Simple LMM with small sample sizes can provide unbiased estimates of fixed effects with minimal inflations in type I error [48]. Because this is a one-arm study, the fixed effect of time and session were the parameters of interest. LMMs specified a random intercept. We used restricted maximum likelihood estimation (REML) to deal with missing data according to an intent-to-treat (ITT) philosophy that is robust against common patterns of missing data. Participants completing at least one assessment were included in the ITT analysis and their feasibility and outcome data were subsequently analyzed.

Following normality verification, nonparametric Friedman repeated measures analysis of variance by rank was used to examine changes in HR across the different time periods during the drug cue-exposure paradigm (periods: baseline, cue-exposure, early craving meditation, and late craving meditation). Post hoc pairwise comparisons between time periods were conducted using Wilcoxon signed rank tests.

### 2.6. Ethics

The study was registered in clinicaltrials.gov in September 2021 (NCT05034276). The research was approved by the University of Utah Investigational Review Board (approval number: IRB_00141495) and study procedures complied with the Helsinki Declaration. Participants provided informed consent and were compensated up to $250 for completing all study activities.

## 3. Results

### 3.1. Sample characteristics

The 28 participants who completed at least one assessment were included in the final analytic sample (four participants who signed consent forms did not complete any assessments). Of those, 22 identified as male (78%), 5 as female (18%), and 1 as non-binary (4%), with a mean (SD) age of 38.5 (9.7) years. Most participants (85%) self-identified as Non-Hispanic/Non-LatinX, and 93% identified as White. Regarding education, approximately half of participants had completed some college (54%). Participant’s income was between $25 and 50K (29%) or below $25K (29%). Regarding MOUD treatment, 25 (89%) participants received buprenorphine and 3 (11%) received methadone (Table 1). The mean duration of MOUD was 2.09 (SD = 2.50) years.

**Table 1.** Participant demographics.

Measure	ITT sample (*n* = 28)
Age	38.5 ± 9.7
Gender	
Male	22 (78%)
Female	5 (18%)
Non-binary	1 (4%)
Race	
White	26 (93%)
Black	1 (4%)
Asian	1 (4%)
Native American / Alaskan	1 (4%)
Ethnicity	
Non-Hispanic / Non-LatinX	24 (85%)
Hispanic / LatinX	1 (4%)
Declined to answer	3 (11%)
Education	
Some high school	1 (4%)
High school graduate or GED	6 (22%)
Some college	15 (54%)
2-yr degree	2 (7%)
4-yr degree	3 (11%)
PhD / MA+	1 (4%)
Income	
< $25,000	8 (29%)
$25,000–50,000	8 (29%)
$50,000–75,000	8 (29%)
$75,000–125,000	3 (11%)
> $125,000	1 (4%)
MOUD	
Buprenorphine	25 (89%)
Methadone	3 (11%)

### 3.2. Feasibility and engagement

**Figure 2.**
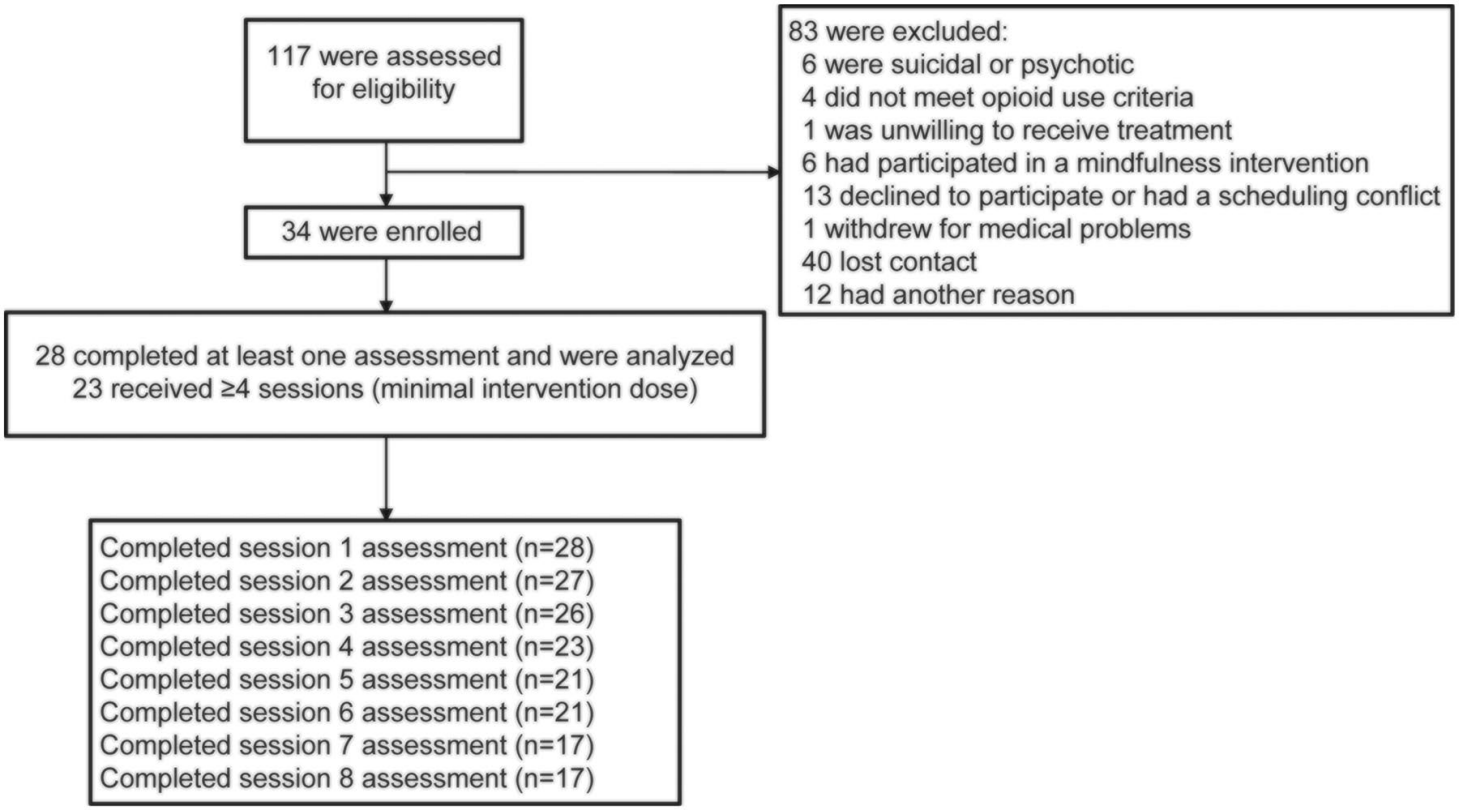
CONSORT diagram.

We screened 117 patients for eligibility; 34 (29%) participants were enrolled. Eighty-three screened patients were excluded: 17 (21%) did not meet eligibility criteria, 25 (30%) declined to participate due to scheduling conflicts or other reasons, 40 (48%) were lost to contact, and one (1%) withdrew due to medical problems. Out of the 34 participants enrolled in the study, 28 completed at least one assessment, 23 (67.5%) completed four or more sessions (the minimal intervention dose), and 17 (50%) completed all intervention sessions. The completion rate among subjects who had completed at least one session, 23 out of 28, was 82%. The CONSORT diagram is presented in Figure 2.

### 3.3. Acceptability, usability and safety

The Net Promoter Score (NPS) was 71.43 (promoters: 78.57%, passive: 14.29%, detractors: 7.14%), indicating a very high level of satisfaction with the intervention. In terms of acceptability, participants’ mean reported usability score of each MORE-VR session was 8.8 (SD = 1.4). Two participants (5.9%) reported non-serious adverse events: one reported a panic attack 20 min after completing session 5, which required medical assistance; and another reported a single episode of nausea during one of the MORE-VR sessions.

### 3.4. Days of opioid use

**Figure 3.**
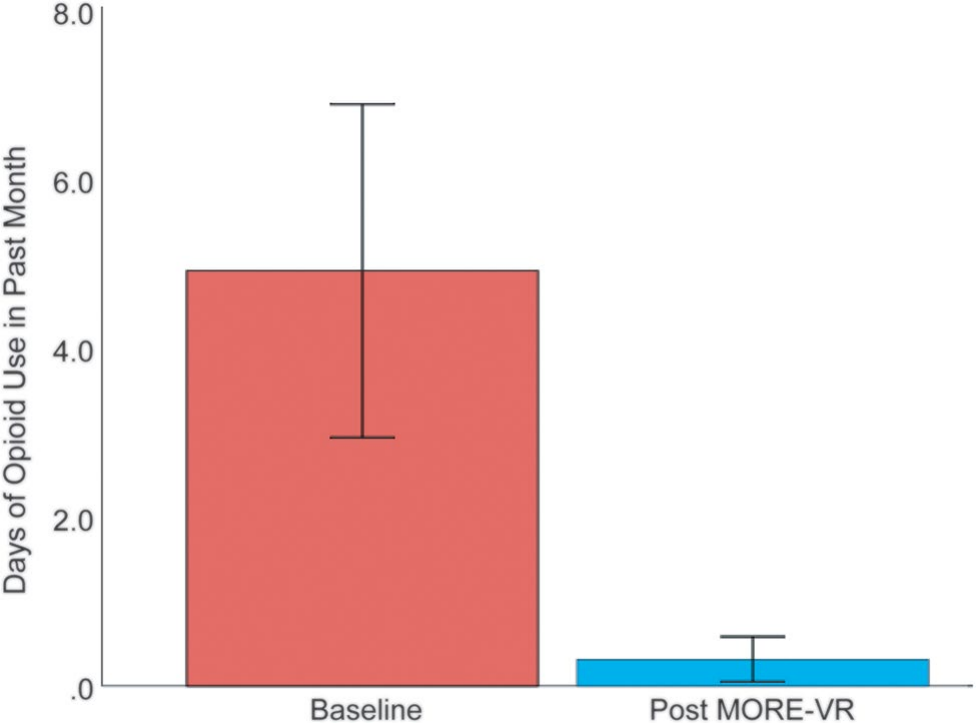
Days of opioid use before and after treatment with MORE-VR.

Participants in MORE-VR reported significantly fewer days of illicit opioid use in the past month at the post-treatment assessment (*M* = 0.33, SE = 0.28) than at pre-treatment (*M* = 5.39, SE = 2.38, *F* = 4.4, *p* = 0.04) (Figure 3).

### 3.5. Affect

**Figure 4.**
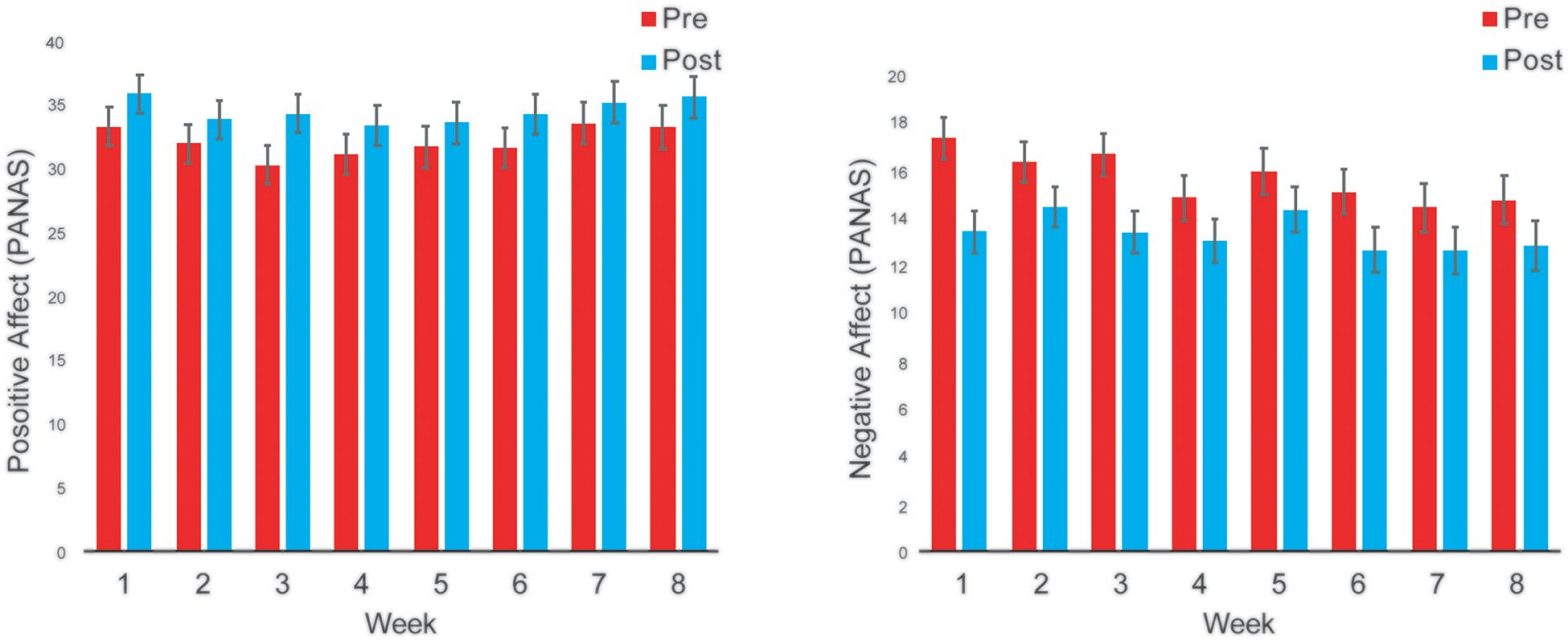
Positive (left) and negative (right) affect scores.

Participants reported a significant increase in positive affect from pre- to post-session as measured by the PANAS (within-sessions *F* = 23.6, *p*<.001) and by NRS (within-sessions *F* = 61.57, *p*<.001). Participants also reported a significant decrease in negative affect on the PANAS (within-sessions *F* = 36.3, *p*<.001). Neither PANAS-positive affect scale (between-sessions *F* = 2.02, *p*=.052), PANAS-negative affect scale (between-sessions *F* = 2.00, *p*=.054) (Figure 4), nor the positive affect NRS (between-sessions *F* = 1.47, *p* = 0.17) showed significant change between sessions over time. The Session X Time interactions for PANAS-positive (*F* = 0.31, *p* = 0.95), positive NRS (*F* = 1.09, *p*=.037), and PANAS-negative (*F* = 0.68, *p* = 0.69) were non-significant, indicating that the impact of MORE-VR sessions on affect did not vary over time.

### 3.6. Craving

**Figure 5.**
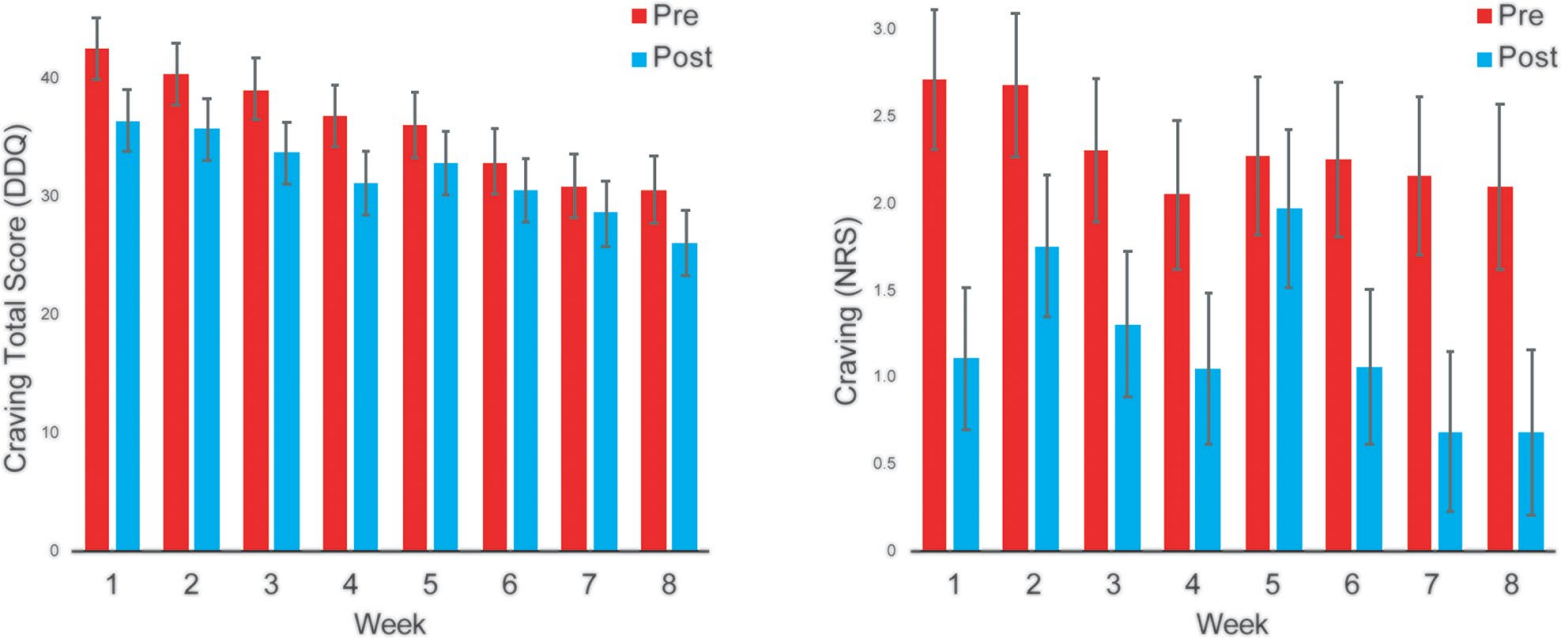
Craving scores on the Desires for Drug Questionnaire (left) and the numeric rating scale (right).

Participants in MORE-VR reported a significant decrease in opioid craving from pre- to post-session as measured by the DDQ (within-sessions *F* = 39.31, *p*<.001), and by NRS (within-sessions *F* = 46.24, *p*<.001). Participants also reported a significant linear decrease in opioid craving across the weekly sessions as measured by the DDQ (between-sessions *F* = 14.98, *p*<.001). The reduction across sessions did not reach statistical significance when measured by the NRS (between sessions *F* = 1.68, *p*=.11) (Figure 5). The Session X Time interaction was non-significant for the DDQ (*F* = 0.62, *p* = 0.74) and NRS (*F* = 0.78, *p* = 0.61), indicating that the impact of MORE-VR sessions on craving did not vary over time.

### 3.7. Heart rate

HR data from seven participants could not be recorded due to technical problems with smartwatch data integration. The Friedman test revealed a significant effect of time period on HR F(3, 37)=6.844, *p* = 0.001. Pairwise comparisons using Wilcoxon signed rank tests (W) revealed that HR increased significantly from the baseline to the drug cue-exposure period (*W* = 21, *p* = 0.049), suggesting an effect of drug cue-exposure on HR reactivity. Then HR decreased significantly from drug cue-exposure to the late mindfulness of craving period (*W* = 21, *p* < 0.001), but not to the earlier period (*W* = 27, *p* = 0.119). HR during the early and late mindfulness of craving periods was not significantly different from the baseline period (early: *W* = 45, *p* = 0.67; late: *W* = 24, *p* = 0.078), suggesting HR returned to baseline levels during the late phase of the mindfulness of craving technique. Finally, HR decreased significantly from the early to the late mindfulness of craving period (*W* = 6, *p* = 0.001) (Supplementary Figure 2).

## 4. Discussion

This study evaluated the acceptability, feasibility, and preliminary efficacy of MORE-VR, a self-guided digital therapeutic for OUD. Results demonstrated an encouraging completion rate, with high acceptability and usability. Analysis of outcomes suggested that MORE-VR could potentially augment the effect of MOUD in reducing opioid use and craving, and increasing positive affect.

With regard to the engagement and feasibility of the MORE-VR intervention, rates of retention in MORE-VR were in line with initial expectations based on prior face-to-face MORE and digital health studies. A large majority of participants completed the 4-session minimal intervention dose. Retention rates were comparable to other clinical studies evaluating psychosocial treatments for opioid use, showing an attrition rate of 42% [49]. Retention rates in the present study were similar to those in previous MORE RCTs conducted in primary care clinics [26,46], and higher than the only MORE study conducted in patients receiving MOUD [27]. Similarly, other MBIs for opioid use disorders have shown completion rates between 57 and 72% [50,51]. A recent feasibility study assessing a 24-weeks mobile-based cognitive behavioral counseling intervention delivered as an adjunct to buprenorphine observed a 64% treatment completion rate [52], which is consistent with the 8-session completion rates reported in the present study. MORE-VR also performed better in terms of retention than most mental health apps reporting average attrition rates of 23% to 48% [53,54]. In sum, retention and completion rates of MORE-VR were consistent with the attrition reported by in-person interventions and other digital therapeutics. Regarding feasibility, a large majority of screened patients (85%) met inclusion criteria, of which 29% were enrolled in the study and 24% completed at least one MORE-VR session. Such an enrollment rate is consistent with other studies assessing VR and digital therapeutics for substance use and chronic pain [55–58].

Despite being a proof-of-concept prototype, participants rated ease of use for MORE-VR highly and reported a very low incidence of adverse events. A large majority of participants would likely recommend MORE-VR to a friend or colleague with OUD. The Net Promoter Score (NPS) yielded a score of 71, indicating a very high level of satisfaction and bringing MORE-VR in line with other digital therapeutics aimed at improving patient care [59–61]. Overall, high acceptability and usability ratings underscored the satisfaction of participants with MORE-VR as an adjunct intervention for OUD.

Regarding symptom outcomes, participants in MORE-VR reported a significant decrease in days of opioid use at the end of the study, and a decrease in opioid craving both within MORE-VR sessions and over time. MORE-VR was also associated with a significant within-session increase in positive affect, a key element of hedonic well-being. Results are consistent with previous face-to-face MORE studies showing a significant decrease in opioid use/misuse [26–28] and meta-analytic data showing that MORE has a medium effect-size in craving reduction [25]. Similarly, multiple trials have demonstrated that MORE enhances positive emotional processes in opioid users [19,47,62] by enhancing neurophysiological responsiveness to natural rewards [19,22,23]. The present study corroborates these findings and supports the notion that MORE can be delivered with high fidelity using a scalable self-guided VR app without the direct involvement of a trained clinician.

HR results indicate that VR presentation of opioid cues during MORE-VR’s cue-exposure exercise elicited significant autonomic drug cue-reactivity, a known marker of craving [63]. Subsequent mindfulness practice coupled with real-time biofeedback was associated with a decrease of HR during drug cue-exposure. These findings suggest that like the face-to-face intervention [19,23], MORE-VR may enhance regulation of drug cue-reactivity and modulate the salience of drug-related cues, thus showing potential as a VR cue-exposure therapy (CET) tool. Nonetheless, future studies should confirm these findings using control conditions to rule out alternative explanations to the observed HR reduction (e.g. habituation) and conduct repeated exposures to assess the extinction of stimulus-response associations.

The study had several limitations as expected from a feasibility study testing a prototype with patients. The lack of a control group precluded any strong inference about the efficacy of MORE-VR. A comparison with treatment-as-usual or a MOUD-only group would be required to assess MORE-VR’s effect on opioid use, craving, or other outcomes. Further, changes in craving and affect may be partially accounted for by expectations arising from the use of a novel digital intervention; to control for this confounding, future clinical trials might employ a sham VR comparator. Moreover, future clinical trials aimed at establishing the efficacy of MORE-VR should evaluate other outcomes such as the retention in MOUD treatment, and control for concurrent interventions that could impact on the efficacy of MORE-VR. The lack of long-term follow-up assessment in the present Phase 1 study did not allow for examination of the potential effects that MORE-VR may have had on MOUD retention and clinical outcomes, or vice versa. Future studies should include more frequent assessments and long-term follow-ups to evaluate treatment trajectory, maintenance effects, and the impact of MORE-VR on MOUD treatment. It is also worth noting that the sample size was small, and ethnically and racially homogeneous. Finally, future studies should test the feasibility, acceptability, and efficacy of MORE-VR in other substance use disorders or addictive behaviors.

## 5. Conclusions

The present feasibility study serves as the foundation for a subsequent Phase 2 RCT assessing the efficacy and safety of MORE-VR compared to treatment-as-usual, and the development of an immersive digital therapy for OUD and other substance use and addictive behaviors. The current study showed promise for MORE-VR as an acceptable and potentially effective adjunct digital therapeutic for people with OUD. MORE-VR might be used as a more cost-effective alternative to in-person psychosocial interventions for OUD requiring significant human interaction by trained clinicians, as well as offer a scalable solution to existing barriers to access evidence-based mental health treatment.

## Supplementary Material

Supplemental Material

## Data Availability

The data that support the findings of this study are available from the corresponding author, ELG., upon reasonable request.
