# Supplementary material for: Virtual reality-based Mindfulness-Oriented Recovery Enhancement (MORE-VR) as an adjunct to medications for opioid use disorder: a Phase 1 trial
Source: Ann Med. 2024 Aug 22;56(1):2392870. doi: 10.1080/07853890.2024.2392870 (PMC11342816; doi:10.1080/07853890.2024.2392870)
Supplement: Supplemental Material [file IANN_A_2392870_SM2749.zip › Suppl_Data/Supplementary Table 1 (9).docx]

**Supplementary Table 1**. Core components of MORE-VR in each lesson

| **Session** | **Psychoeducational Topic** | **Mindfulness and Therapeutic Components (Duration)** |
| --- | --- | --- |
| **1** | Mindfulness of stress and emotional pain | - Introduction to stress and mindfulness (10:50). - Body scan practice (10:04). - Intro to Mindful breathing: Using mindfulness to identify the maladaptive cognitive-emotional reactions (11:11). - Homework assignment (2:04) |
| **2** | Mindfulness of automaticity | - Mindful breathing (5:34) - Automaticity and addiction education (6:48) - Exposure and mindfulness: virtual food exercise and awareness of automatic habits (10:26) - Homework assignment (4:22) |
| **3** | Reappraisal of maladaptive thoughts | - Mindful breathing (15:20) - Introduction to cognitive reappraisal (8:29) - Mindful reappraisal: reframe maladaptive thoughts about recent stressors (7:17) - Education on Relapse (3:30) - Homework assignment (1:34) |
| **4** | Savoring natural rewards | - Mindful breathing (5:34) - Mindful savoring education (7:08) - Mindful savoring exercise: Savor a virtual rose (12:48) - Homework assignment (0:47) |
| **5** | Mindfulness of craving | - Mindful breathing (5:34) - Education on craving (7:07) - Craving exercise: virtual cue-exposure to opioids and paraphernalia and mindfulness (20:36) - Homework assignment (1:04) |
| **6** | Disrupting the link between stress, & craving | - Mindful breathing (5:34) - Education on stress response and craving (5:38) - Stress exposure exercise and relaxation: envisioning a stressful life situation and relaxation (20:33). - Homework assignment (0:47) |
| **7** | Mindfulness to meaning and interdependence | - Mindful breathing (15:20) - Education on interdependence (6:30) - Meditation on interdependence: contemplating meaning in life and interconnectedness (13:25) - Homework assignment (0:47) |
| **8** | Developing a mindful recovery plan | - Mindful breathing (15:20) - Program reflection: Identifying addiction relapse triggers and skills from the MORE-VR to address each trigger (8:38) - Future visualization and maintaining mindfulness practice exercise (12:52) |
