# Supplementary figures and images for: Virtual reality-based Mindfulness-Oriented Recovery Enhancement (MORE-VR) as an adjunct to medications for opioid use disorder: a Phase 1 trial
Source: Ann Med. 2024 Aug 22;56(1):2392870. doi: 10.1080/07853890.2024.2392870 (PMC11342816; doi:10.1080/07853890.2024.2392870)

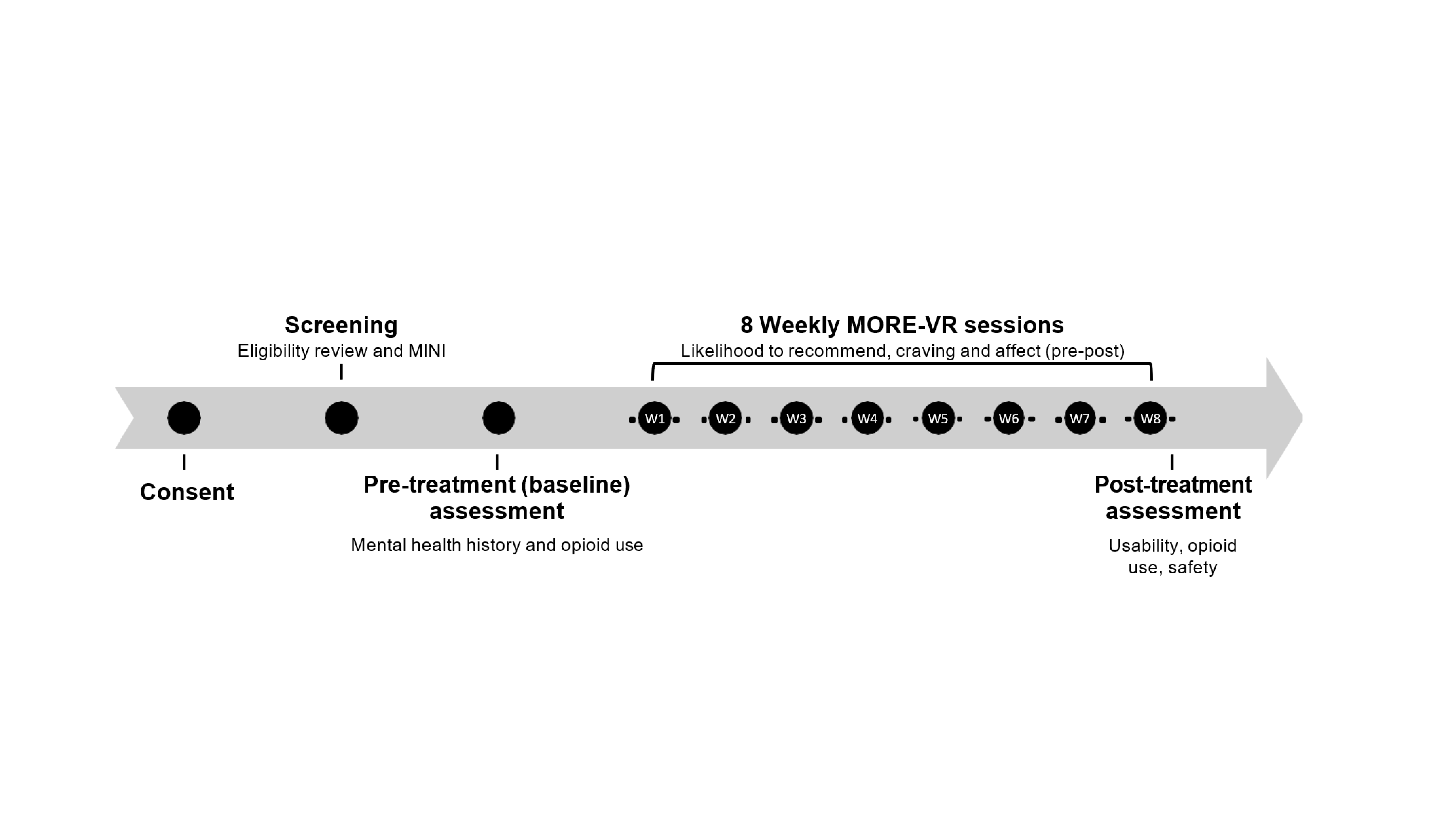

Supplement: Supplemental Material [file IANN_A_2392870_SM2749.zip › Suppl_Data/Supplementary Figure 1 REV.tif]

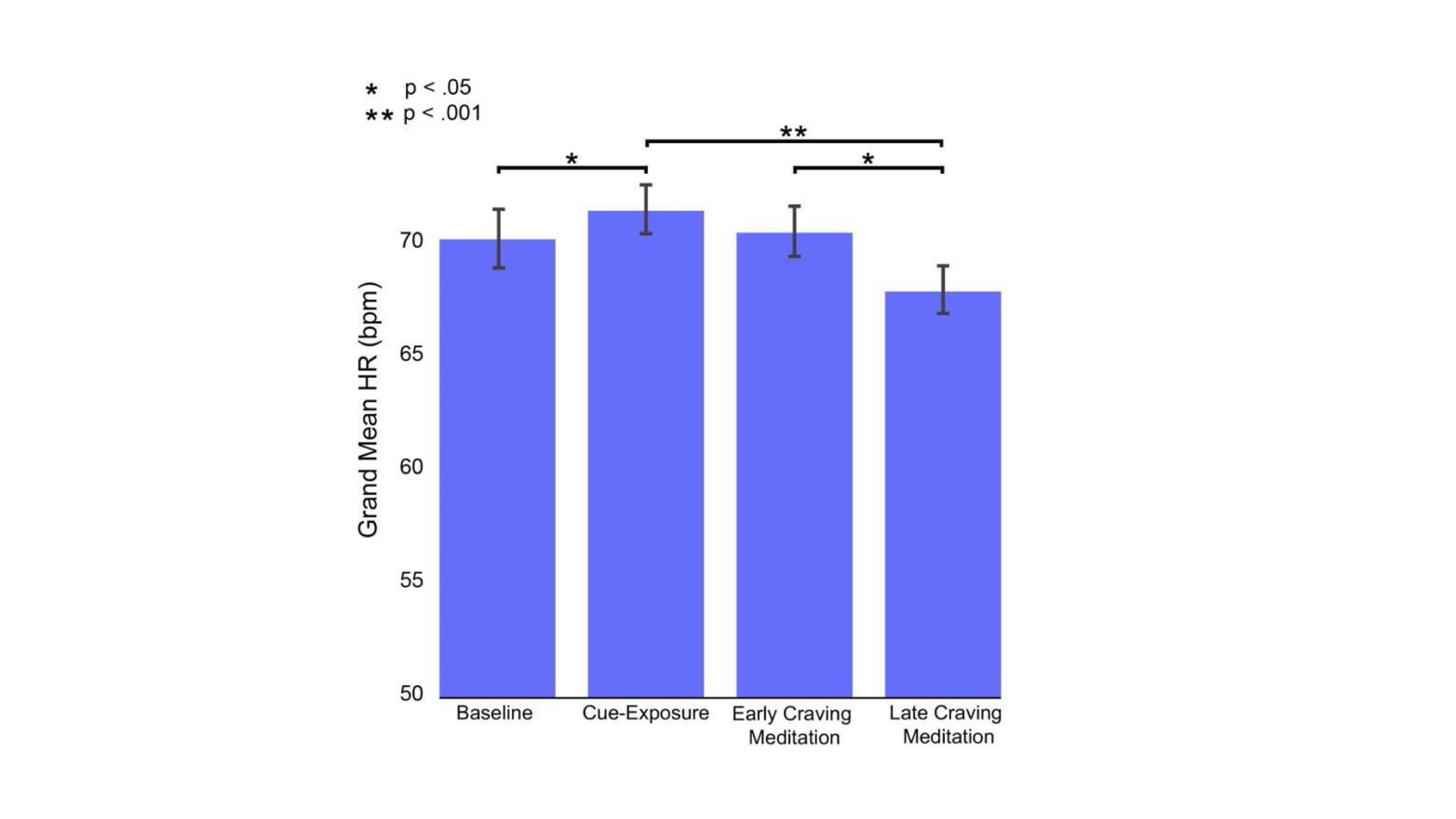

Supplement: Supplemental Material [file IANN_A_2392870_SM2749.zip › Suppl_Data/Supplementary Figure 2.tif]
